# Bismuth-213 for Targeted Radionuclide Therapy: From Atom to Bedside

**DOI:** 10.3390/pharmaceutics13050599

**Published:** 2021-04-21

**Authors:** Stephen Ahenkorah, Irwin Cassells, Christophe M. Deroose, Thomas Cardinaels, Andrew R. Burgoyne, Guy Bormans, Maarten Ooms, Frederik Cleeren

**Affiliations:** 1 Institute for Nuclear Materials Science, Belgian Nuclear Research Center (SCK CEN), 2400 Mol, Belgium; stephen.ahenkorah@sckcen.be (S.A.); irwin.cassells@sckcen.be (I.C.); thomas.cardinaels@sckcen.be (T.C.); andrew.burgoyne@sckcen.be (A.R.B.); 2 Radiopharmaceutical Research, Department of Pharmacy and Pharmacology, University of Leuven, 3000 Leuven, Belgium; guy.bormans@kuleuven.be; 3 Nuclear Medicine Unit, University Hospitals Leuven, 3000 Leuven, Belgium; christophe.deroose@uzleuven.be; 4 Nuclear Medicine and Molecular Imaging, Department of Imaging and Pathology, University of Leuven, 3000 Leuven, Belgium; 5 Department of Chemistry, University of Leuven, 3001 Leuven, Belgium

**Keywords:** bismuth-213, targeted radionuclide therapy, targeted alpha therapy, radiopharmaceutical, bifunctional chelator, vector molecule

## Abstract

In contrast to external high energy photon or proton therapy, targeted radionuclide therapy (TRNT) is a systemic cancer treatment allowing targeted irradiation of a primary tumor and all its metastases, resulting in less collateral damage to normal tissues. The α-emitting radionuclide bismuth-213 (^213^Bi) has interesting properties and can be considered as a magic bullet for TRNT. The benefits and drawbacks of targeted alpha therapy with ^213^Bi are discussed in this review, covering the entire chain from radionuclide production to bedside. First, the radionuclide properties and production of ^225^Ac and its daughter ^213^Bi are discussed, followed by the fundamental chemical properties of bismuth. Next, an overview of available acyclic and macrocyclic bifunctional chelators for bismuth and general considerations for designing a ^213^Bi-radiopharmaceutical are provided. Finally, we provide an overview of preclinical and clinical studies involving ^213^Bi-radiopharmaceuticals, as well as the future perspectives of this promising cancer treatment option.

## 1. Introduction

**Figure 1:**
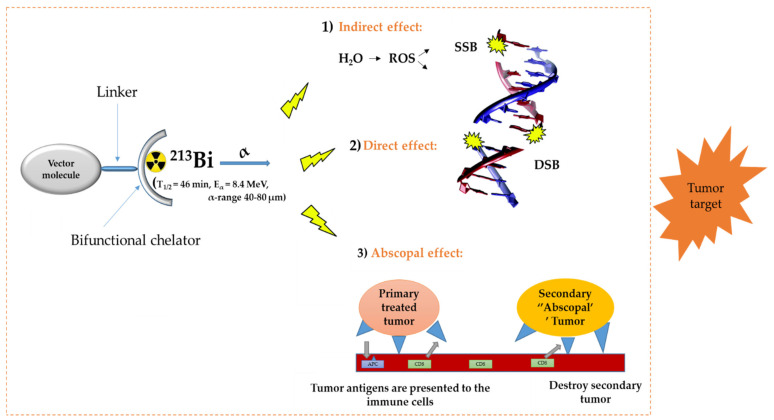
General concept of TRNT. ROS = reactive oxygen species, SSB = single strand break, DSB = double strand break, APC = antigen presenting cells, CD6 = cluster of differentiation 6. The DNA structure was reproduced from Gill and Vallis 2019, with permission from the Royal Society of Chemistry [9], Royal Society of Chemistry, 2019.

In the diagnosis, monitoring, and treatment of cancer patients, nuclear medicine plays an important role. Radiopharmaceuticals consist generally out of three functional elements: (1) a vector molecule (or carrier) that shows high selectivity and affinity for a target; (2) a radionuclide; and (3) a linker or chelator to attach the former to the latter (Figure 1) [1,2,3,4]. A fundamental and critical component of a radiometal-based radiopharmaceutical is the chelator, the ligand that binds the radiometal ion in a stable coordination complex so that it can be properly directed to its molecular target in vivo [5,6,7]. The radiopharmaceutical is mostly distributed within the body by the vascular system and allows targeting of a primary tumor and all its metastases. The specific decay characteristics of the radionuclide attached to the vector molecule determine if the radiopharmaceutical can be used for diagnostic (molecular imaging) or therapeutic (TRNT, targeted radionuclide therapy) purposes. The goal of targeted radionuclide therapy is to deliver sufficiently high doses of ionizing radiation to specific disease sites for cure, disease control, or symptomatic (e.g., pain of hormonal secretion) palliation. The biological effect of radionuclide therapy is obtained by three mechanisms: (1) Interaction of ionizing radiation with water in which chemically active free radicals (reactive oxygen species, ROS) are formed that can react with biomolecules (phospholipids, proteins, RNA, DNA, etc.), thereby irreversibly damaging the cells. (2) Direct interaction of ionizing radiation with DNA in which single-strand (SSB), double-strand (DSB), or cluster breaks can occur. (3) During treatment, a phenomenon called the abscopal effect can occur when radiation reduces not only the targeted tumor but also leads to the shrinkage of untreated tumors elsewhere in the body. Though the exact biological mechanisms accountable for the abscopal effect are yet to be identified, the immune system is considered the major player in this significant role [1,8].

Auger-, beta minus (β^–^-), and alpha (α-) emitters are the three main subgroups of therapeutic radionuclides. α-decay is a radioactive decay in which an α-particle, consisting of two protons and two neutrons, is emitted from the nucleus. Tumor cells can be selectively destroyed, and the healthy tissue is minimally damaged due to the short penetration depth of α-particles (40–80 µm, corresponding to 2–10 cell diameter). An α-particle is characterized by a high-energy linear transfer (LET, 100 kEV/µm) and thus a high relative biological effect (RBE). The high LET of α-particles results in a high rate of double-strand and cluster DNA breaks and consequently irreparable damage, producing the high kill rate of α-emitting radioisotopes both in normoxic as well as in a hypoxic tumor cell environment, which is known to be more resistant to photon and electron-based irradiation [10]. Therefore, targeted alpha therapy (TAT) can be considered as promising cancer treatment, especially suitable for the treatment of tumors with small diameters and which have a spatially homogeneous expression of the molecule targeted by the vector. Sufficient expression of the target in the malignant tissue must be confirmed before TAT can be initiated. This can be done by first performing a positron emission tomography (PET) or single photon emission computed tomography (SPECT) scan, after injection of the diagnostic sister of the therapeutic radiopharmaceutical. If the diagnostic radiopharmaceutical can truthfully predict the pharmacokinetics of its therapeutic sister, the diagnostic scan also allows accurate calculation of the dosimetry. This class of radiopharmaceuticals are called theranostic agents [11].

It is also important to mention here that the theranostic approach could also involve a single radionuclide agent—this theory stipulates the use of a single radionuclide, producing both therapeutic radiation and a low-energy γ-ray for SPECT to deliver dosimetric data. Examples of some of these radionuclides are ^47^Sc (T_½_ = 3.35 d) and ^67^Cu (T_½_ = 2.58 d) [12].

The α-emitting radionuclide ^213^Bi (*T*_1/2_ = 45.6 min, E_α_ = 8.4 MeV, γ = 440 keV, α-particle range = 40–80 µm) has interesting properties and might be considered as a magic bullet for TRNT [5,9]. However, like every cancer treatment modality, ^213^Bi-based TAT has advantages and disadvantages. Indeed, ^213^Bi can be considered as powerful precision ammunition, but it needs to be handled with care. In function of the target, the most suited vector molecule needs to be selected and the biological half-life of the vector should be compatible with the 45.6 min physical half-life of ^213^Bi, which is substantially shorter than any therapeutic radionuclide in current routine clinical use. Further, an appropriate chelator needs to be carefully chosen to match the physical properties of ^213^Bi. 

In this review, we will discuss advantages and disadvantages of TAT using ^213^Bi, covering the entire chain from radionuclide production to bedside. This includes production of the mother isotope ^225^Ac and the use of the ^225^Ac/^213^Bi generator, chemical and physical properties of ^213^Bi, an overview of acyclic and macrocyclic chelating agents that are available for ^213^Bi, general considerations for designing a ^213^Bi-radiopharmaceutical, and an in-depth overview of non-clinical and clinical studies that have been performed, discussing dosimetry and toxicity issues. Lastly, we will discuss the future perspectives of this intriguing potential cancer treatment.

## 2. Radionuclide Properties and Production of ^225^Ac and Its Daughter ^213^Bi

### 2.1. Decay Properties of ^225^Ac and ^213^Bi

**Figure 2:**
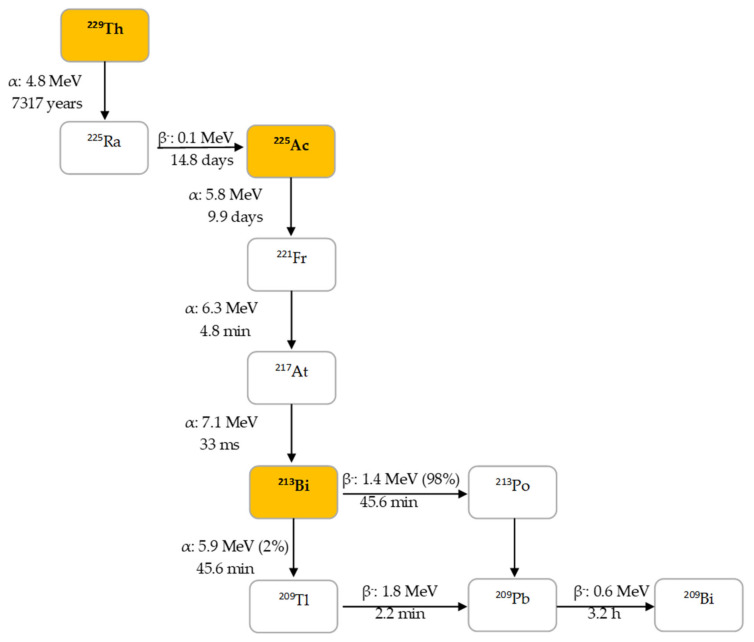
Decay chain of thorium-229 to ^225^Ac and ^213^Bi.

^225^Ac is the parent radionuclide of ^213^Bi and is a relatively long-lived α-emitter with a half-life of 9.9 d. It decays via a cascade of six short-lived radionuclide daughters to stable ^209^Bi with four net alpha particles emitted per decay (Figure 2). ^213^Bi has a half-life of 45.6 min and shows branching decay (β^–^ and α decay), and most of the α-particles emitted originate from the β^–^ branch (see Figure 2). Indeed, it mostly decays via β^–^ emission to the short-lived α-emitter ^213^Po (*T*_1/2_ = 4.2 µs, E_α_ = 8.375 MeV, 97.8%, Figure 2). The residual 2.2% of ^213^Bi decays leads to ^209^Tl (E_α_ = 5.549 MeV, 0.16%, E_α_ = 5.869 MeV, 2.0%). Finally, ^213^Po and ^209^Tl decay via ^209^Pb (*T*_1/2_ = 3.25 h, β^–^) to stable ^209^Bi [13]. 

In human tissue, the α-particle released by ^213^Po (E_α_ = 8.375 MeV) has a path length of 85 µm. It is this radionuclide that produces >98% of the α-particle energy released per disintegration of ^213^Bi and could be considered as the radionuclide that provides ^213^Bi cytotoxicity. The bulk of the total particle energy released per disintegration of ^213^Bi comes from α-decay, accounting for 92.7%, while 7.3% comes from β-particle emission, which includes the decay of ^209^Pb [14]. The decay of ^213^Bi is followed by the emission of a 435 keV-photon (98% abundance) that could potentially be used for SPECT with a gamma camera equipped with high energy collimators, permitting detailed evaluation of the biodistribution of ^213^Bi in vivo [15].

It is noteworthy to mention that high activity ^225^Ac/^213^Bi generators are required to allow production of clinical amounts of ^213^Bi. Optimal clinical injected activities depend on the vector molecules; for prostate-specific membrane antigen (PSMA)-targeting radiopharmaceuticals, this has been determined to be 4–8 MBq of ^225^Ac [16,17]. In contrast, to have the same number of alpha-particles emitted, 5–10 GBq is needed for ^213^Bi-labeled radiopharmaceuticals [18]. This was estimated based on the Equations (1) and (2) below and the fact that ^225^Ac delivers four α particles in its total decay scheme, while ^213^Bi delivers net only one α particle. However, this is a rough estimation that does not consider the biological clearance of the radioprobes. Definitely, the much shorter half-life of ^213^Bi compared to the half-life of ^225^Ac is the key reason for the high activity levels that are needed when using ^213^Bi for targeted radionuclide therapy (Equation (2)).

*A* = λ·*N* (1)

λ = ln(2)/*T*_1/2_ (2)

where *A* = activity (Bq, disintegrations per second), *N* = number of atoms, λ = decay constant.

### 2.2. Current Strategies for ^225^Ac Production

**Figure 3:**
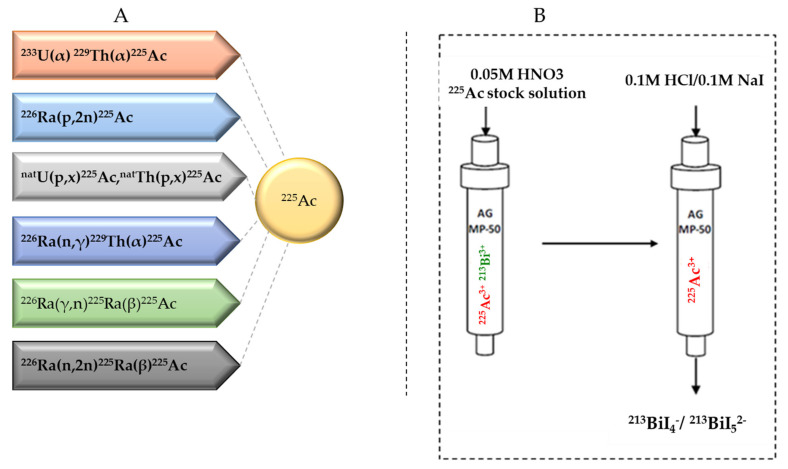
(**A**) Production routes for ^225^Ac and (**B**) the ^225^Ac/^213^Bi generator.

Several approaches for ^225^Ac and ^213^Bi production have been recently reviewed and discussed in detail (Figure 3) [7,13]. The most utilized strategy is the radiochemical extraction of ^225^Ac from ^229^Th (*T*_1/2_ = 7317 years) sources originating from the decay of fissile ^233^U. This results in “carrier free” ^225^Ac using the generator method, but the available ^233^U stocks and thus ^229^Th stocks are limited because ^233^U management is restricted by the requirements concerning non-proliferation of fissile materials. Most of the ^213^Bi and ^225^Ac used in clinical tests and research activities worldwide has so far been produced by this approach [19]. Two examples of ^229^Th sources that can produce clinically relevant activities of ^225^Ac are (1) the US Department of Energy, Oak Ridge National Laboratory (ORNL) in Oak Ridge, TN, United States of America and (2) the Directorate for Nuclear Safety and Security of the JRC of the European Commission in Karlsruhe, Germany. Additionally, at the Belgian Nuclear Research Centre (SCK CEN) in Mol, Belgium, very pure sources of ^229^Th were identified, processed, and used for pre-clinical studies [20]. The total global annual ^225^Ac production volume is approximately 55–65 GBq [21] and cannot meet the growing demand for ^225^Ac, but interestingly, it has been reported that stocks will increase by extraction of additional ^229^Th from US legacy wastes [7].

Cyclotron production is an alternative method for ^225^Ac production. Medium-energy proton irradiation of ^226^Ra using the reaction ^226^Ra(p,2n)^225^Ac is considered to be a viable approach. The advantage of this method is the widespread availability of appropriate cyclotrons worldwide, especially in Europe [22,23], and high amounts of clinical doses of ^225^Ac could be produced in a reliable way. It is important to mention that medical cyclotrons used to produce radioisotopes (15–25 MeV) are considered to be feasible for basic and applied research [24]. The downside with this approach is that ^226^Ra is not an easy isotope to work with due to the presence of the highly radiotoxic noble gas ^222^Rn in its decay chain. Therefore, it is unlikely that this will ever enter a clinical cyclotron.

Further, high-energy proton irradiation (0.6–2 GeV) of uranium and thorium spallation targets via the reaction ^nat^U(p,x)^225^Ac is another approach for the production of ^225^Ac. The existence of several suitable high-energy proton facilities makes this an achievable prospect. However, such accelerator-produced ^225^Ac contains a low percentage (0.1–0.3%) ^227^Ac (*T*_1/2_ = 21.77y) at the end of bombardment, which might be a serious limitation in terms of clinical translation and waste management [25,26]. Nevertheless, ^225^Ac obtained through high-energy accelerators could be entirely appropriate for ^225^Ac/^213^Bi generator production (see 2.3), even with co-production of ^227^Ac, as all actinium species will be retained on the generator. 

Another production path being explored is transmutation of ^226^Ra to ^229^Th by an intense neutron flux. This will lead to three successive neutron capture reactions: ^226^Ra(n,γ)^227^Ra, ^227^Ac(n,γ)^228^Ac, and ^228^Th(n,γ)^229^Th. However, production of many orders of magnitude of ^228^Th (*T*_1/2_ = 1.9 y) intermediate, and handling of the radium target remains a challenge [18].

^226^Ra(γ,n)^225^Ra is another reaction path for ^225^Ac production that has been determined experimentally [27,28]. This strategy explored irradiation of old radium needles with high-energy X-rays from electron linear accelerators (linacs).

Finally, transmutation of ^226^Ra to ^225^Ra by fast neutrons via the reaction ^226^Ra(n,2n)^225^Ra is also under consideration. It is noteworthy to point out that the limitation for all the strategies discussed is the handling of the radium targets. However, earlier results with these methods have been promising [22].

To conclude, all clinical studies with ^225^Ac/^213^Bi radiopharmaceuticals were performed to date with ^225^Ac originating from ^229^Th stocks, but other accelerator-based production routes were heavily investigated in the last decade. This progress will hopefully assure reliable production and delivery of ^225^Ac to radiopharmacy institutes in the near future, allowing more preclinical research and multicenter clinical trials with both ^225^Ac and ^213^Bi-radiopharmaceuticals.

### 2.3. ^225^Ac/^213^Bi Radionuclide Generators

^225^Ac can be loaded on ^225^Ac/^213^Bi generators to deliver ^213^Bi on site, but it can also be used directly as a therapeutic radionuclide. ^225^Ac/^213^Bi generators are well explored and discussed [29,30,31]. The most established strategy is based on the direct generator method, in which the parent ^225^Ac in acidic solution (e.g., 0.05M HNO_3_) is strongly retained by the sorbent (e.g., AG MP-50 cation exchange resin) and ^213^Bi is eluted. Elution is performed generally with a mixture of 0.1M HCl/0.1M NaI to obtain ^213^Bi in the form of ^213^BiI_4_^-^ and ^213^BiI_5_^2-^ that can be directly used for radiochemistry purposes. (Figure 3) [7]. These generators can potentially be used clinically due to the relatively long parent half-life, which allows shipment of the generator to radiopharmacy facilities at long distance. Additionally, the transient equilibrium of the ^225^Ac-^213^Bi permits elution at approximately every 3 h [32]. These generators can provide weeks of reliable in-house generation of ^213^Bi for radiolabeling purposes [33]. High activity (up to 4 GBq ^225^Ac) generator systems, developed at JRC Karlsruhe, have been reported with yields of ^213^Bi elution exceeding 80% and low breakthrough of ^225^Ac of less than 0.2 ppm [32].

## 3. Fundamental Chemistry of Bi

Bi is an element that in comparison to the lighter main group metals has received only limited attention in the past. However, Bi compounds are or were used in a wide spectrum of applications ranging from non-toxic pigments and catalysts to biocompatible additives in dental materials and remedies in human and veterinary medicine [34,35]. To select a good chelator for ^213^Bi to be used in radiopharmaceutical applications, it is important to have an in-depth knowledge about the chemical properties of Bi.

The Bi atom has 83 electrons distributed among different energy levels. Electrons occupying the highest and outermost energy levels are responsible for its chemical reactivity [34]. Bi has a [Xe] 4f^14^5d^10^6s^2^6p^3^ electron configuration and tends to form trivalent bismuth (Bi^3+^) with 6s^2^ valence configuration. For this tendency of the 6s^2^ electron pair to remain formally unoxidized in Bi compounds (i.e., core-like nature of the 6s electrons), the term “inert pair effect” [36], or “nonhybridization effect” has often been used [37]. Bi has two major oxidation states (III and V), with Bi(III) being the most common and stable oxidation state. However, rare occurrences of Bi(II) and Bi(IV) have been reported [38].

The aqueous Bi chemistry is predominantly dominated by Bi(III) and, unlike the d-block metals, it forms complex and clustered structures across the pH scale [39,40]. At low pH (e.g., pK_a1_ = 1.5), Bi(III) readily undergoes hydrolysis to form Bi(III) hydroxide [41]. In dilute aqueous solutions ([Bi] ˂ 1 × 10^−5^ molarity), mononuclear complexes predominate considerably over a wide range of pH values [42], and these mononuclear complexes of Bi largely follow a stepwise addition of OH^–^ such that Bi(OH)_x_^(3−x)^ (where x ≤ 4) complexes are formed sequentially with increasing pH. 

Bi(III) is a borderline metal ion, according to Pearson’s hard–soft acid–base (HSAB) theory [43]. Nevertheless, with considerable affinity for nitrogen and oxygen donor atoms [44,45,46], some researchers have reported Bi(III) as a strong Lewis acid [39]. This property makes complexation with amine donor groups possible even at low pH [47]. The complexation kinetics with N- and O-donor atoms is often fast, with equilibrium achieved in a few minutes [47]. Some amino ligand binding constants have predictive nature, which can be expressed as logK_1_(polyamine) = 1.152 logβ_n_(NH_3_) + (n−1) × 1.744, where β_n_ refers to the stability of the complex containing primary amine analogues and n is the number of primary amine analogues of the polyamine [47]. As observed with aqua ligands, small O-donor ligands bridge multiple Bi atoms to form cluster species [48].

In Bi coordination chemistry, Bi(III) complexes exhibit a variety of coordination numbers, with values between 3–10 [41]. Low coordination numbers can be well-predicted using valence shell electron pair repulsion theory (VSEPR, see Table 1) [41]. Nonetheless, higher coordination numbers (>6) require more complex levels of theory to account for structural influence on geometry (e.g., vibrational coupling, etc.).

## 4. Bifunctional Chelating Ligands for ^213^Bi

When a radiometal is used for targeted radionuclide therapy, a chelator with an extra reactive functional group is required. This moiety, also called a bifunctional chelator (BF), guarantees a covalent connection with the vector molecule and forms a stable radiometal complex. Because of the high cost of the α-emitting radionuclides, short physical half-life in the case of ^213^Bi, and radioprotection reasons, quantitative yields using fast and mild radiolabeling conditions (e.g., room temperature, 5 min) are desired to facilitate efficient good manufacturing practice (GMP)-compliant on-site production of ^213^Bi radiopharmaceuticals. Further, if the vector molecule is a heat-sensitive biomolecule or peptide, mild aqueous labelling conditions should be applied to avoid degradation of the vector molecule. Importantly, the radiometal–chelator complex should have a high thermodynamic stability and kinetic inertness to avoid the in vivo dissociation that typically occurs via transchelation to serum proteins and enzymes. Several papers have reported that naked ^213^Bi, dissociated from its chelating ligands, tends to accumulate in the kidney [5,49,50]. In this chapter, we will give an overview of most important bifunctional chelators currently used for Bi^3+^.

### 4.1. DTPA and DTPA-Derivatives

The discovery of acyclic chelators with rapid radiometal ion coordination kinetics, high in vivo stability, and kinetic inertness has become a requirement for TRNT due to the emergence of antibody vectors.

**Figure 4:**
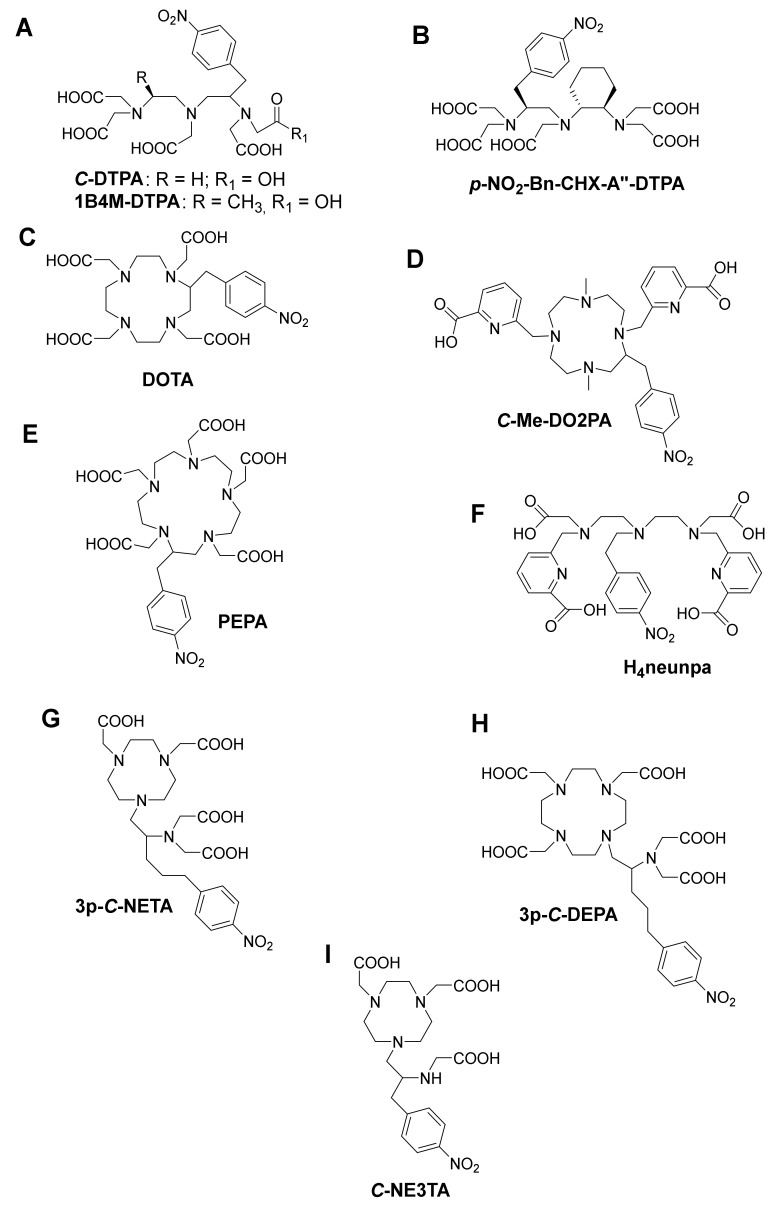
Some structures of bifunctional chelators currently used for ^213^Bi (**A**) DTPA and 1B4M-DTPA (**B**) CHX-A’’-DTPA, (**C**) DOTA, (**D**) Me-DO2PA, (**E**) PEPA, (**F**) H4neunpa, (**G**) 3p-*C*-NETA (**H**) 3p-*C*-DEPA and (**I**) *C*-NE3TA.

As one of the oldest chelators used in radiopharmaceuticals, BF diethylenetriamine-*N,N,N’,N’’,N’’*-pentaacetic acid (DTPA, Figure 4A) can rapidly radiolabel different radiometal ions, even at room temperature [3]. However, in vivo stability remains the major limitation, and radiometal DTPA complexes are generally less stable as the corresponding complexes with DOTA (1,4,7,10-tetraazacyclododecane-1,4,7,10-tetraacetic acid, Figure 4**C**). BF-DTPA has been used successfully with the FDA-approved SPECT agent ^111^In-DTPA-octreotide (OctreoScan^TM^), a somatostatin receptor targeting peptide-conjugate, used for imaging neuroendocrine tumors [3,51]. Additionally, it has been successfully radiolabeled with different radiometals, such as ^213^Bi, ^111^In, ^177^Lu, ^64^Cu, and ^86/90^Y, but has been made superfluous by the introduction of more stable DTPA-derivatives such as 1B4M-DTPA and BF-CHX-A’’-DTPA (Figure 4B) [2,3]. BF-1B4M-DTPA (Figure 4A), a DTPA derivative that contains a single methyl group on one of its ethylene backbones, has been successfully used for the FDA-approved ^90^Y therapeutic immunoconjugate ibritumomab tiuxetan (BF-1B4M-DTPA, Zevalin^®^) [52]. The presence of the cyclohexyl moiety of CHX-A’’-DTPA provides the chelator with additional rigidity and imposes a significant degree of preorganization on the metal ion binding site, augmenting kinetic inertness, but impeding radiolabeling kinetics as compared with DTPA [3].

While kidney uptake caused by in vivo dissociation of ^213^Bi from the chelator is problematic with ^213^Bi-DTPA constructs, [53] a slight improvement is recorded with ^213^Bi(1B4M-DTPA). Current ^213^Bi TAT research mostly uses CHX-A”-DTPA (logK_ML_ = 34.9–35.6) [53,54], as the Bi complex has shown significantly improved inertness compared with Bi-DTPA. A clear backbone rigidity effect on ^206^Bi release was observed after a study comparing renal uptake of B72.3-mAb radiolabeled with ^206^Bi using DTPA, CHX-A’’-DTPA, and 1B4M-DTPA as chelating ligands. Kidney uptake amounted to 27.2%ID/g for DTPA, 7.8%ID/g for CHX-A’’-DTPA, and 13.2%ID/g for BF-1B4M-DTPA, respectively [55]. CHX-A’’-DTPA displayed considerably superior stability over DTPA; however, its stability could still not match that of DOTA.

### 4.2. DOTA and DOTA-Derivatives

**Figure 5:**
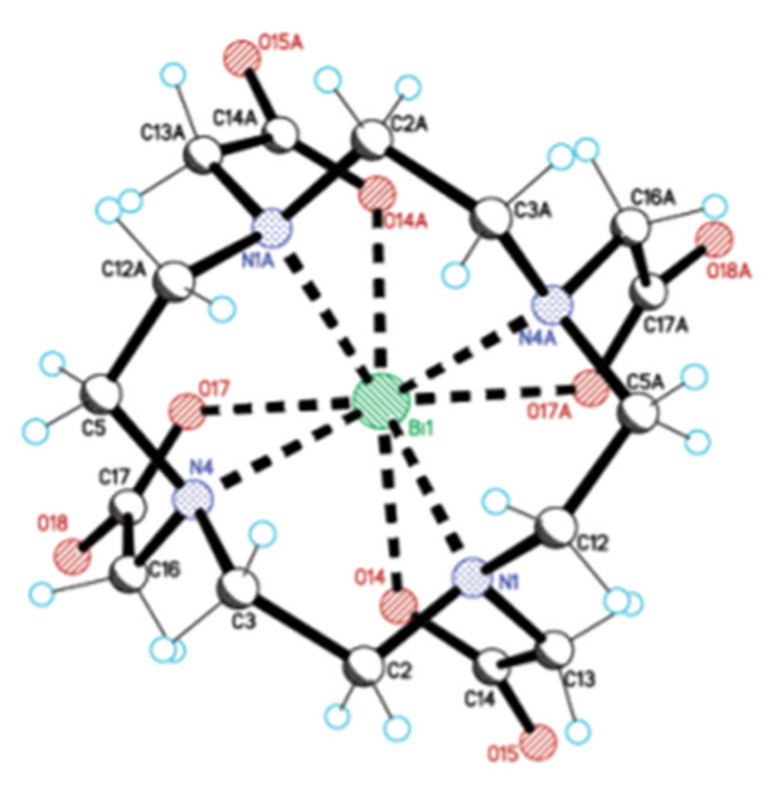
Crystal structure of NaBiDOTA·H_2_O. Reprinted (adapted) from E. Brücher et al., 2003, with permission from [58]; American Chemical Society, 2003.

The current “gold standard” BF chelator for ^213^Bi is the amino carboxylate macrocycle DOTA, and ^213^Bi-DOTA bioconjugates have been reported to be stable in vitro and in vivo for at least two hours [56,57]. Bi^3+^ adopts a square antiprism geometry with DOTA in the [Bi-DOTA]-complex (see Figure 5) [58]. Despite the relatively high thermodynamic stability of the ^213^Bi-DOTA complex (logK_ML_ = 30.3), DOTA has several drawbacks as a ^213^Bi chelator. DOTA’s typical radiolabeling conditions require heating at high temperatures (e.g., 30–60 min at 95 °C) at pH 4–9, depending on the type of buffer used. Additionally, it has been demonstrated that a high concentration of DOTA (10 µM) is required to achieve quantitative yields for ^213^Bi-labeling. In contrast, for CHX-A’’-DTPA, a concentration of 1 µM is in general sufficient to achieve quantitative yields [59]. The relatively short half-life of ^213^Bi requires fast radiolabeling, which is not the case for DOTA and results in significant loss of radioactivity due to decay. Additionally, the high temperatures are unsuitable for heat-sensitive proteins of interest for TRNT, as this may cause the proteins to denature.

BF-PEPA (Figure 4E), a chelator that is an expanded version of DOTA, has also been studied with ^205/206^Bi. This chelator was developed to improve the association kinetics of Bi-DOTA radiocomplexes. Unfortunately, this chelator was discovered not to be ideal for Bi because a lower tumor uptake and increased kidney uptake of ^205,206^Bi-B3-PEPA was observed compared with the corresponding CHX-A’’-DTPA complex [50].

Me-DO2PA (Figure 4D), a [12] aneN4 bearing two picolinic acid arms and two methyl-capped amines, has also been reported to be stable in vivo when radiolabeled with ^213^Bi [60,61].

### 4.3. NETA and DEPA-Derivatives

NETA ({4-[2-(bis-carboxymethyl-amino)-ethyl]-7-carboxymethyl- [1,4,7] triazonan-1-yl}-acetic acid, Figure 4 G) and DEPA (7-[2-(bis-carboxymethyl-amino)-ethyl]-4,10-bis-carboxymethyl-1,4,7,10-tetraazacyclododec-1-yl-acetic acid, Figure 4H) are promising chelators for radionuclide therapy of α- and β^–^-emitters, including ^90^Y, ^177^Lu (*T*_1/2_ = 6.7 days), ^206^Bi, ^207^Bi, ^213^Bi, and ^212^Pb. NETA possesses both a parent macrocyclic NOTA (1,4,7-triazacyclononane-*N,N’,N’’*-triacetic acid) backbone and a flexible acyclic tridentate pendant arm (iminodiacetic acid). DEPA on the other hand is made up of a donor system incorporating both macrocyclic DOTA and an acyclic tridentate pendant arm (iminodiacetic acid). The idea of designing NETA/DEPA was to integrate the advantage of both the macrocyclic and acyclic frameworks, i.e., rapid complexation (favorable formation kinetics) at ambient temperatures with radionuclides and high thermodynamic stability [62,63].

A ^205/206^Bi-NETA analogue displayed a lower degree of degradation compared with ^205/206^Bi-CHX-A′′-DTPA after in vitro challenge experiments. Biodistribution experiments of unconjugated [^205/206^Bi(*C*-NETA)]- in non-tumor-bearing mice showed a high level of kidney retention (24.63 ± 2.79%ID/g) after one hour. However, a biodistribution study of the seven-coordinate analogue [^205/206^Bi(*C*-NE3TA)] (Figure 4I) showed reduced kidney retention (4.69 ± 0.55%ID/g), even though having a seemingly non-coordinatively saturated Bi^3+^ metal center [62,64]. A version of NETA with a prolonged linker between the coordinating functional groups and pendant isothiocyanate group (*p*-SCN-Bn) in the subsequent experiments stipulates that the instability could originate from coupling group interference. 3p*-C*-NETA-trastuzumab showed faster labeling of ^205/206^Bi compared with DOTA-trastuzumab, and an in vivo experiment on mice bearing subcutaneous tumors (LS-174T, which expresses HER-2, the target of trastuzumab) demonstrated significant tumor accumulation without increasing kidney uptake (after 24 h) [65]. This study showed that 3p-*C*-NETA could be radiolabeled at room temperature in quantitative yields and have high stability in vitro and in vivo, which was not the case with DOTA.

The ^205/206^Bi-DEPA complex has also been studied to show similarly promising results with DTPA as benchmark [66,67]. After 72 h p.i., ^205/206^Bi-*C*-DEPA-trastuzumab remained 100% intact in human serum. However, in the case of ^205/206^Bi-DTPA-trastuzumab, only 77% was intact after 72 h [66]. ^205/206^Bi-3p-*C*-DEPA-trastuzumab also showed significant tumor uptake in tumor-bearing (LS-174T) mice. As suggested by Price and Orvig, an in-depth assessment of NETA versus DEPA for Bi-based bioconjugates might be useful to identify the “gold standard” of Bi chelators [3]. Finally, a promising bifunctional picolinic acid-based scaffold chelator, H4neunpa (Figure 4F), has also been studied. The only concern is that the stability of the Bi^3+^ complex (log K_ML_ = 28.8) was lower than that of DOTA and DTPA; however, the pM value is the similar to that of DOTA (pM = 27), providing potential for in vivo use [68]. Complex geometry and thermodynamic parameters of ^213^Bi with different chelators are summarized in Table 2.

## 5. General Considerations for Designing a ^213^Bi-radiopharmaceutical

In order to realize the potential and favorable properties of ^213^Bi, specifically targeted carriers need to be developed [1,3,4]. Indeed, the vector molecule is an essential part of the therapeutic radiopharmaceutical because it is responsible for the selective interaction with the target, leading to a high concentration of the radionuclide in the target tissue. The target of interest should have sufficiently high differential expression in the target tissue versus the background tissue, combined with sufficiently high absolute expression (B_max_). Several targets are currently being investigated for TAT with ^213^Bi-labeled probes, such as human epidermal growth factor receptor 2 (HER2) [69], cluster of differentiation 20 (CD20) [70], CD33 [71,72], CD45 [73], substance P [74,75,76], somatostatin receptors [19], prostate-specific membrane antigen (PSMA) [77], and epidermal growth factor receptor (EGFR) [78].

Due to reversible interactions, such as an affinity-based receptor or a transporter binding, the retention in the target tissue can be governed by equilibrium association and dissociation. However, the tracer can be internalized and kept in the cell after binding to the target. This internalization can maximize selective irradiation of the tumor since it leads to pseudo-irreversible kinetics.

Radiopharmaceuticals are usually administered intravenously, distributed quickly over the body, and are concentrated in the target tissue or cells due to physical or chemical interactions. In the surrounding tissue, which lacks the interaction mechanism, the concentration of the radiopharmaceutical is in equilibrium with the plasma concentration that is reduced in function of time because of clearance from plasma by excretory organs such as kidneys and liver. Renal clearance and urinary excretion are preferred over hepatobiliary clearance for oncological applications for TAT with ^213^Bi since hepatobiliary clearance results in a slow transfer through the gastro-intestinal tract, resulting in undesired high levels of abdominal activity.

**Figure 6:**
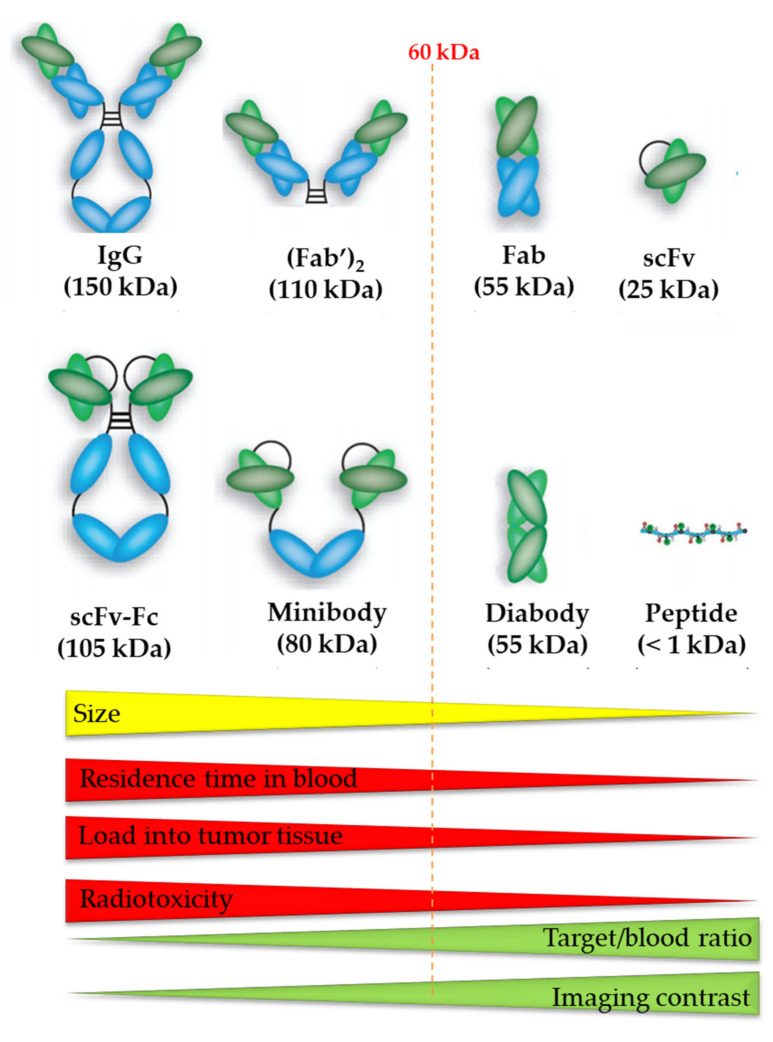
The size of the vector molecule determines the residence time of blood, tumor accumulation, radiotoxicity, target-to-blood ratio, and imaging contrast of the radiopharmaceutical. Image was adapted from Hong H. et al., 2008 and modified with permission from [79], SAGE Journals, 2008.

The vector molecule can consist of a small organic molecule, a peptide, or a protein including antibodies and antibody fragments (Figure 6) [4]. It is important that the vector molecule has a high affinity and selectivity for the target and maintains this affinity and selectivity after conjugation with the radionuclide. As ^213^Bi is a radiometal, derivatization of small organic molecules with a bulky chelator usually significantly alters its binding properties. Therefore, mostly peptides and antibody fragments are used as vector molecule in ^213^Bi-labeled radiopharmaceuticals. Their large size and the presence of non-binding pockets usually make them less sensitive to changes in target affinity when being derivatized with bulky bifunctional ligands.

An important factor when assessing the therapeutic potential of vectors is the kinetic profile of the carrier. In general, vector molecules with longer circulation time in blood have the highest tumor accumulation, which is beneficial for the efficacy of TAT treatment. On the other hand, longer residence time in blood also involves unavoidable higher radiation dose to healthy tissues. Therefore, the half-life of the radionuclide should be compatible with the plasma half-life of the vector to ensure a sufficient high tumor/background ratio. One should note that the selectivity for radiation damage to malignant tissue is potentially higher for longer-lived radionuclides, which is beneficial in a therapeutic setting. In this respect, the short physical half-life of ^213^Bi is a disadvantage when combined with a carrier molecule with a long plasma half-life. Accumulation of such a carrier in the tumor tissue is too slow in comparison with the half-life of ^213^Bi, resulting in less selective irradiation of the tumor tissue. In contrast, ^213^Bi is a much better match with vector molecules that have a short biological half-life, such as some small molecules, several peptides, and antibody fragments (including nanobodies). These molecules accumulate rapidly in the tumor tissue, and therefore allow ^213^Bi to deposit its dose to the tumor before it is fully decayed. The TAT studies with ^213^Bi-PSMA-617 in a patient with metastasized castration-resistant prostate cancer that was refractory to ^177^Lu-radiotherapy illustrates the potential of the combination of ^213^Bi with a vector molecule with short biological half-life [80]. Further, an in vitro and in vivo preclinical study with a ^213^Bi-labeled nanobody for TAT showed promising results (see Table 3) [5].

Although carriers with a short plasma half-life are preferred, several ^213^Bi-labeled vectors with slow kinetics have been reported. One way to bypass the kinetic incompatibility is local administration. Locoregional delivery of ^213^Bi-radiopharmaceuticals compared to systematic administration has the potential to significantly increase efficacy, while minimizing systemic toxicity to non-targeted tissues. A disadvantage is that it is no longer a systemic treatment and that not all metastases will be treated, except if after locoregional injection there is substantial spill-over to the systemic circulation (e.g., injection in the hepatic artery). With locoregional delivery, the short-lived radionuclide ^213^Bi can be combined with vector molecules with a long biological half-life, such as mAbs, as only high binding affinity to the target is important and not the plasma pharmacokinetic properties of the radiopharmaceutical.

Pretargeted radiotherapy is another approach for combining a vector with long plasma half-life with a radionuclide with a short half-life, such as ^213^Bi [99]. First, a (slow) tumor-accumulating vector molecule carrying a tag is administered systemically. Once accumulated at the target sites and largely cleared from the blood, a radiolabeled agent that rapidly recognizes the tag of the tumor-bound vector in vivo and is cleared fast from plasma is injected. Upon encountering the targeting vector, fast and efficient bio-orthogonal ligation will take place between the two molecules, which leads to the in vivo formation of the final radioconjugate, resulting in specific irradiation of target tissue and low radiation burden of healthy tissue.

In the following two chapters non-clinical and clinical experience with ^213^Bi-labeled probes will be discussed, organized by the type of vector molecule.

## 6. Preclinical TAT Studies with ^213^Bi-labeled Probes

### 6.1. Antibodies

Monoclonal antibodies (mAbs), with their impeccable affinity for tumor antigens, have become powerful tools in the diagnosis and treatment of cancer, particularly when combined with therapeutic agents such as radionuclides and cytotoxic drugs, thanks to advances in hybridoma technology in the 1980s [87,100]. A mAb has the potential to bind antigen epitopes with high affinity, including tumor-related antigens, and are interesting vector molecules for TRNT because of high tumor accumulation (Figure 6) [4]. A variety of preclinical investigations have been conducted using mAbs labeled with ^213^Bi as radiometal (Table 3).

TRNT performed with 3.7 MBq of ^213^Bi-labeled 9E7.4 anti-CD138 mAb increased median survival to 80 days, compared with 37 days for the untreated control, and resulted in 45% cure in a multiple myeloma mice model. β^–^-TRNT performed with 18.5 MBq of ^177^Lu-labeled 9E7.4 mAb was well tolerated and increased mouse survival significantly (54 vs. 37 days in the control group); however, the authors reported that no mice were cured with this treatment [87].

Another study has shown that fractionated intravesical TRNT with ^213^Bi-anti-EGFR-mAb is a promising approach in advanced bladder carcinoma. Therapeutic efficacy was evaluated via overall survival and toxicity toward normal urothelium by histopathological analysis. Mice without treatment and those treated with the native anti-EGFR-mAb showed median survivals of 65.4 and 57.6 d, respectively. After fractionated treatment with 0.93 MBq, ^213^Bi-anti-EGFR-MAb animals lived for an average of 141.5 d, with 33% survival for at least 268 d. The animals survived for an average of 131.8 d after fractionated treatment with 0.46 MBq ^213^Bi-anti-EGFR-mAb, with 30% survival for more than 300 d. It was concluded that no toxic side effects on the normal urothelium were observed, even after treatment with 3.7 MBq of ^213^Bi-anti-EGFR-mAb [81].

### 6.2. Antibody Fragments

Recent advances in bioengineering have led to the development of antibody fragments—such as Fab (50 kDa), F(ab’)_2_ (110kDa), 25-kDa single-chain Fv (scFv), diabodies (55 kDa), nanobodies (15kDa), and minibodies (80 kDa)—without affecting their affinity and specificity [101]. Smaller mAb derivatives are transported more rapidly to the tumor site and can penetrate the tumor more effectively. Because of their smaller size and lack of an Fc region, they are swiftly cleared from circulation, resulting in high tumor-to-background ratios at early time-points (Figure 6). Successful conjugation of ^213^Bi to anti-HER2 C6.5 scFv and diabody molecules has been reported. However, there was no tumor-specific therapeutic effect, which was most likely due to the in vivo instability of the scFv and diabody molecules. The physical half-life of ^213^Bi, 45.6 min, was found to be too short for the systemically administered diabody to accumulate sufficiently in the solid tumor [102].

For the first time, a ^213^Bi-labeled HER2-sdAb nanobody was successfully produced and characterized in vitro and in vivo in a preclinical setting. ^213^Bi-DTPA-2Rs15d sdAb exhibited high in vivo stability and specific accumulation in target tissue after i.v. administration in mice. When administered in therapeutic doses, ^213^Bi-DTPA-2Rs15d sdAb increased the median survival of mice (80 d compared with 56 d in the control group), particularly when used in combination with trastuzumab (140 d). These findings suggest that ^213^Bi-DTPA-sdAb could be used as a new radioconjugate for TAT, both alone and in combination with trastuzumab, to treat HER2^+^ metastatic cancer. Authors indicate it might be interesting to evaluate ^213^Bi-DTPA-2Rs15d sdAb in a trastuzumab-resistant tumor model. To conclude, the rapid accumulation of ^213^Bi-labeled 2Rs15d sdAb in HER2-expressing tumors demonstrates that nanobodies are promising vector molecules in combination with the short-lived ^213^Bi [5].

### 6.3. Peptides

Peptides have been used extensively in nuclear medicine for peptide receptor radionuclide therapy (PRRT). In the randomized NETTER-1 clinical trial, treatment with ^177^Lu-DOTATATE resulted in markedly longer progression-free survival and a significantly higher response rate than high-dose octreotide LAR among patients with advanced midgut neuroendocrine tumors [103]. Peptides are designed by rational methods to bind with high specificity and selectivity to their target cells. Due to their ease of synthesis using chemical or molecular biological techniques, peptide sequences can also be easily modified [104]. Oncogenic protein sequences, structures, and pattern interactions are all readily accessible, allowing peptides to be engineered specifically for TAT. Peptides have several important advantages over proteins or antibodies as vector molecule for TRNT: they are small, mostly stable, easy to synthesize, they can be modified to further increase the metabolic stability, and it is straightforward to site-specifically attach a bifunctional chelator, allowing radiolabeling. Furthermore, they are typically less immunogenic than recombinant antibodies or proteins [105].

An in vitro comparison of ^213^Bi- and ^177^Lu-based PRRT has been performed. Absorbed doses up to 7 Gy were obtained by 5.2 MBq ^213^Bi-DOTATATE, and the majority of the dose was caused by α-particle radiation. The cell survival of BON or CA20948 cells after exposure to ^213^Bi-DTPA and ^213^Bi-DOTATATE showed a linear-exponential relationship with the absorbed dose, confirming the strong LET character of ^213^Bi. CA20948 demonstrated the standard curvature of the linear-quadratic model after exposure to ^177^Lu-DOTATATE and the reference ^137^Cs. With ^213^Bi-DOTATATE, 10% CA20948 cell survival was achieved at 3 Gy, which is six times less than the 18 Gy needed for ^177^Lu-DOTATATE and less than the 5 Gy required after ^137^Cs external exposure [56].

Important progress has been made in the production and application of ^213^Bi-optimized vehicles so far. Despite the promising preliminary results, there is still much room for improvement, especially in the development of new coupling chemistries and the elucidation and optimization of in vivo biodistribution.

## 7. Clinical TAT Studies with ^213^Bi-labeled Radiopharmaceuticals

It is suggested that TAT is ideally suited for hematologic malignancies due to the easy and fast accessibility of malignant cells in blood, bone marrow, lymph nodes, and spleen, as well as their typical high radiosensitivity [32,70]. The majority of clinical trials for hematologic malignancies using α-particle therapy have focused on acute myeloid leukemia (AML) [71,72]. Nonetheless, preclinical studies have shown activity against other cancer types, including non-Hodgkin’s lymphoma and multiple myeloma [92,106].

To date, ^213^Bi-labeled radiopharmaceuticals has been used to treat more than 200 patients for leukemia, lymphoma, melanoma, bladder cancer, glioma, and neuroendocrine tumors [7]. The two main approaches currently used for the administration of this radiopharmaceutical are locoregional and systemic administration.

### 7.1. Locoregional Administration

#### 7.1.1. Intravesical TRNT

A locoregional pilot study to evaluate the feasibility, tolerability, and efficacy of ^213^Bi-anti-EGFR mAb treatment in patients with bacillus Calmette-Guerin (BCG) refractory carcinoma in situ (CIS) was performed [78]. A single intravesical instillation (or two instillations) of 366–821 MBq of ^213^Bi-anti-EGFR immunoconjugate (with molar activities in the range of 0.37–0.82 GBq/mg) was tolerated without any adverse effects. Four patients showed a complete response, i.e., no visible CIS, eight weeks after the first or second treatment. Three of the four patients were still tumor-free at the time of the publication, i.e., three months after the second treatment and 30 and 44 months after the first treatment. However, since this was a pilot study, the authors concluded that additional studies are required to confirm this therapeutic outcome [78].

#### 7.1.2. Intracerebral Substance-P PRRT

Interest in PRRT has steadily grown because of the advantages of targeting cellular receptors in vivo with highly tumor-specific targeting and high tumoral activity concentration. Unparalleled attempts to develop radiolabeled receptor-binding somatostatin analogues for the treatment of neuroendocrine tumors have occurred in recent decades, and these efforts have facilitated the evolution of PRRT and paved the way for the development of other receptor-targeting peptides [103,107,108,109]. The highest number of patients treated with locoregional administration of ^213^Bi was gathered for the treatment of grade II to IV glioma with radiolabeled DOTA-Substance P analog (substance-P binds to GPCR neurokinin type 1 receptor). However, its short plasma half-life presented a challenge to researchers. In a clinical study, ^213^Bi-DOTA-Substance P was administered locoregionally into the brain tumor or tumor cavity via an implanted catheter. Patients were treated with up to 14.1 GBq ^213^Bi-DOTA-Substance P, administered in up to eight treatment cycles at two-month intervals. No severe side effects were recorded. The median survival time from the start of ^213^Bi-DOTA-Substance P was 7.5 months. Several patients showed complete remissions, and no recurrence was observed up to 23.8 months after the end of therapy. A subgroup analysis indicated prolonged survival times for grade IV patients in comparison with standard treatments [75]. These data should encourage future randomized controlled trials.

#### 7.1.3. Intralesional Melanoma TRNT

Injection of a high concentration of a drug directly into skin lesions without significant systemic absorption is also becoming increasingly popular. The rationale for this technique is the establishment of a subepidermal depot that bypasses the superficial barrier zone.

Intralesional melanoma TRNT with ^213^Bi conjugated to the benign tumor targeting antibody vector 9.2.27 has been studied. Sixteen melanoma patients positive to the monoclonal antibody 9.2.27 were recruited. ^213^Bi-immunoconjugate activities from 5.5 to 50 MBq injected into lesions of different sizes resulted in massive cell death, as observed by the presence of tumor debris. The ^213^Bi-immunoconjugate was very efficient in providing a high radiation dose to the tumor lesions while sparing the surrounding tissues. Blood proteins and electrolytes did not display any major changes after intralesional injection [110].

### 7.2. Systemic Administration

#### 7.2.1. Acute Myeloid Leukemia TRNT

mAb-TAT uses α-emitting radionuclide labeled antibodies directed against a tumor-associated antigen to deliver a lethal radiation dose selectively to tumor cells. Table 4 summarizes the current state of clinical investigation of ^213^Bi-mAb-TAT radiopharmaceuticals.

An initial phase I study conducted in 18 patients with relapsed or refractory acute myeloid leukemia (AML) demonstrated the safety and antitumor effects of ^213^Bi-lintuzumab. Subsequently, ^213^Bi-lintuzumab produced remissions in AML patients after partial cytoreduction with cytarabine in a phase I/II trial [72]. The short half-life of ^213^Bi and the need for an onsite generator presented a major obstacle to the wide-spread use of mAb-TAT with ^213^Bi. Therefore, a second-generation construct was developed using ^225^Ac that was directly conjugated to the antibody. A phase I trial demonstrated that a single infusion of ^225^Ac-lintuzumab could be given safely at doses up to 111 kBq/kg, with anti-leukemic activity across all studied activity levels. In a second phase I study, 28% of older patients with untreated AML and unfit for intensive chemotherapy had objective responses after receiving fractionated ^225^Ac-lintuzumab and low-dose cytarabine [111,112].

#### 7.2.2. SSTR PRRT

The only ^213^Bi PRRT study so far was performed by Kratochwil et al., in 2014. Eight patients with multi-resistant neuroendocrine tumors refractory to therapy with beta emitter labeled ^90^Y-/^177^Lu-DOTATOC were treated with ^213^Bi-DOTATOC. Seven of them underwent intra-arterial injection into the hepatic artery, which results in an enriched exposure of the liver metastases but also allows systemic targeting. Even though the patients presented were in an advanced disease setting and had developed resistance against therapy with beta emitting ^90^Y-/^177^Lu-DOTATOC, ^213^Bi-DOTATOC therapy resulted in a considerable number of long-lasting antitumor responses, including one complete remission [19].

#### 7.2.3. ^213^Bi-PSMA

PSMA has received great attention in nuclear medicine in the last decade, as it is a promising molecular target for small molecules, antibodies, and antibody fragments [113]. A first-in-human treatment with ^213^Bi-PSMA-617 was performed in a patient with mCRPC (metastatic castration-resistant prostate cancer) that was progressive under conventional therapy. The patient was treated with two cycles of ^213^Bi-PSMA-617 with a cumulative activity of 592 MBq. Restaging with ^68^Ga-PSMA PET/CT after 11 months showed an outstanding molecular imaging response. This patient also exhibited a biochemical response (decrease in prostate-specific antigen levels from 237 μg/L to 43 μg/L) [80].

## 8. Future Perspectives of ^213^Bi-TAT

The recent data on the remarkable therapeutic efficacy of ^213^Bi-bioconjugates for cancer therapy have significantly ignited interest in the clinical application of TAT. The implementation of ^213^Bi-bioconjugates does not only offer an auspicious therapeutic option for cancer treatment but also strongly underlines an important potential of the concept of TAT. Data from clinical studies with ^213^Bi-DOTATOC and ^213^Bi-PSMA-617 suggest that patients do not develop resistance to therapy with α-emitters, whereas this is the case with conventional drugs and with β^–^-emitters therapy. TAT could provide a useful additional treatment option to patients who were progressive under conventional treatments. A major advantage of ^213^Bi-TAT compared with ^225^Ac-TAT is that there is no problem of uncontrolled redistribution of recoiled daughter nuclides, which might cause considerable toxic effects to heathy tissues [117]. To successfully translate ^213^Bi-bioconjugate probes from bench-to-bedside, one needs to consider the half-life of ^213^Bi (46 min), which should match the tumor and plasma kinetics of the vector molecule. Evidently, a therapeutic agent that dispels most of its energy before reaching its target will cause more harm than good. Low molecular weight peptide ligands and antibody fragments labeled with ^213^Bi are for this reason promising due to their favorable fast pharmacokinetics.

Additionally, treatment of cancer-associated fibroblasts (CAF), which are highly expressed in the stroma of most tumor entities, is an emerging tumor targeting area. CAFs, in contrast with normal fibroblasts, overexpress fibroblast activation protein α (FAP). Novel radioprobes, both for diagnostic and therapeutic applications, were designed and are based on FAP inhibitors (FAPI) as vector molecule (e.g., ^68^Ga-DOTA-FAPI-46). FAPI PET images are characterized by rapid kinetics, very low background activity (no uptake in brain, muscle, brown fat, bowel, etc.) and high tumor-to-background contrast. The fast pharmacokinetics of FAPI vector molecules might be an ideal match with the short physical half-life of ^213^Bi [118,119,120,121]. Hence, this will be an interesting area to explore for future ^213^Bi radiopharmaceutical development.

For ^213^Bi chelation chemistry, closer inspection reveals that “gold standard” ligands such as DOTA might not be ideal for clinical application. The chelator should clearly match with the chemical properties of ^213^Bi, and the thermodynamic stability should be balanced with the formation kinetics of the Bi-complexes. This is particularly important for the practical preparation of ^213^Bi-radiopharmaceuticals. NETA and DEPA derivatives have so far demonstrated to be an ideal match for ^213^Bi. However, the potential of NETA and DEPA for use in ^213^Bi radiopharmaceuticals requires further investigation before it can be translated to a clinical setting.

Another area to consider is in vivo kidney radiotoxicity, due to kidney uptake and retention of ^213^Bi-radioprobes. The mechanisms responsible for unwanted kidney uptake and retention are well explored. Radioconjugates with a molecular weight of less than 60 kDa (Figure 6) are filtered through the glomerulus and are often reabsorbed and transported to the lysosomal compartment. The long residence time of radionuclides in the kidney causes a high radiation burden for the kidneys. This can lead to tubular necrosis and limits the total therapeutic dose, especially when using the short-lived radionuclide ^213^Bi. Several strategies have been explored to mitigate the nephrotoxic aspect of ^213^Bi-labeled radioprobes during cancer treatment [5,57,122,123], and some of these approaches have been approved for clinical applications. The co-infusion of lysine or the plasma expander Gelofusine^®^ with ^213^Bi-bioconjugates during TAT has been reported to have a 3-fold reduction in kidney uptake and retention [57,124]. Another promising approach could be that ^213^Bi-radioprobes will be developed with a cleavable linker—positioned between the Bi-chelator complex and vector molecule—that is recognized specifically by renal brush border membrane enzymes, so that the radionuclides are excreted more efficiently toward the bladder instead of being retained in the kidneys.

A study has demonstrated that derivatives of [^68^Ga]Ga-DOTA-AmBz-MVK-OH could be cleaved specifically by neutral endopeptidase (NEP), and therefore the amino acids linker MVK could reduce kidney uptake of radiolabeled DOTA-conjugated peptides and peptidomimetics [125]. These findings would allow development of radiometal-based radiopharmaceuticals with clinically relevant lower renal radioactivity levels [126].

To conclude, TAT using ^213^Bi has demonstrated interesting results preclinically and clinically. The limitation is that ^213^Bi radionuclide has a relatively short half-life (46 min). However, it can be made available to hospitals from a ^225^Ac/^213^Bi generator that can be used over a more than 10-day period. ^213^Bi is an ideal match for vector molecules with fast plasma clearance, fast tumor targeting, but limited tumoral retention.

## Figures and Tables

**Table 1:** Summary of geometries for each coordination number. Table was adapted from Sun H. et al., 1997, with permission from [41], John Wiley and Sons, 2006.

Coordination Number	Geometry	Example
3	Pyramidal	Bi(SAr)_3_
4	Trigonal bipyramidal	[Bi{OP(NMe_2_)_3_}_2_][Fe(CO)_2_(η_5_–Cp)_5_F_2_][PF_6_]
5	Square-based pyramidal	Na_2_[Bi(SC_6_F_5_)_5_)](THF)_4_
5	Trigonal antiprism	{Bi(NO_3_)bis[1-azepanyl-4-(2-thieniyl)-2,3-diazapenta-1-3 diene- 1 -thlolato-N^3^,S]}
6	Octahedral	[Bi_6_O_4_(OH_4_)]^6+^
7	Trigonal Dodecahedron	{Bi(NO_3_)bis[1-azepanyl-4-(2-pyridyl)-2,3-diazapenta-1,3 diene-1-thiolato-N’,N^3^,S]}
8	Bicapped trigonal prism	[Bi(nta)(H_2_O)_2_]
9	Tricapped trigonal prism	[Bi(H_2_O)_9_](CF_3_SO_3_)_3_
9	Monocapped square antiprism	(guanidinium)_2_[Bi(dtpa)]·4H_2_O

**Table 2:** Overview of Bi^3+^ Chelators, Complex Geometry, and Thermodynamic Parameters. Table was adapted from Kostelnik et al., 2019, with permission from [2], American Chemical Society, 2019.

Metal Ion	Ligand	Coordinating Nuclei	Geometry	LogK_ML_^b^	pM ^c^
Bi^3+^	DOTA	N_4_O_4_	Square antiprism	30.3	27.0
Bi^3+^	Me-DO2PA	N_6_O_2_	Square antiprism	34.2	28.6
Bi^3+^	DTPA	N_3_O_5_	Square antiprism	33.9–35.2	-
Bi^3+^	CHX-DTPA	N_3_O_5_	Square antiprism	34.9–35.6	-
Bi^3+^	NETA	N_4_O_4_	Square antiprism	-	-
Bi^3+^	DEPA	N_4_O_5_/N_5_O_4_^a^	Distorted dodecahedron	-	-
Bi^3+^	H_4_neunpa	N_5_O_4_	Distorted dodecahedron	28.8	-

^a^ under investigation, ^b^ *K*_ML_ represents the formation constant of the metal complex, ^c^ pM value is the negative log of the concentration of free metal ion uncomplexed by a given chelator under specific conditions.

**Table 3:** Overview of some preclinical studies with ^213^Bi.

Bioconjugate	Key Findings	Cancer Type	Reference
^213^Bi-anti-EGFR-mAb	The animals survived for an average of 131.8 d after fractionated treatment with 0.46 MBq ^213^Bi-anti-EGFR-mAb, with 30% remaining for more than 300 days. Even after treatment with 3.7 MBq of ^213^Bi-anti-EGFR-mAb, no toxic side effects on normal urothelium were observed.	Human bladder carcinoma (local instillation of ^213^Bi-anti-EGFR-mAb)	[81]
^213^Bi-69-11 antibody	Antibody 69-11 localized significantly in pancreatic ductal adenocarcinoma cancer (PDAC) xenografts in mice in vivo and ex vivo. TAT of PDAC xenografts with ^213^Bi-69-11 was effective, safe, and CETN1-specific.	Pancreatic cancer	[15]
^213^Bi-h8C3 antibody	Treatments with anti-PD-1 antibody alone had a modest impact on tumor size, while the combination therapy with ^213^Bi-h8C3 resulted in a substantial slowing of tumor development and improved animal survival.	Melanoma	[82]
^213^Bi-8C3 or ^213^Bi-6D2 antibody	Antibody binding to melanin was shown to be dependent on both charge and hydrophobic interactions, and in vivo evidence supports the development of 8C3 IgG as a radioimmunotherapy reagent for metastatic melanoma.	Melanoma	[83]
^213^Bi-DOTATATE	A 10% cell survival of CA20948 was reached at doses of 3 Gy with ^213^Bi-DOTATATE, a factor six lower than the 18 Gy found for ^177^Lu-DOTATATE and below the 5 Gy after ^137^Cs external exposure.	Pancreatic cancer	[56]
^213^Bi-IMP288-mAb	^213^Bi-IMP288 cleared from the bloodstream rapidly; blood levels were 0.44 ± 0.28% ID/g 30 min after injection. Except for the kidneys, where uptake was 1.8 ± 1.1% ID/g 30 min after injection, uptake in normal tissues was poor.	Colon cancer	[6]
^213^Bi-MX35-mAb	The tumor-free fraction in animals given 3 MBq/mL of ^213^Bi-MX35 was 0.55, while it was 0.78 in animals given 9 MBq/mL of ^213^Bi-MX35. The tumor-free fraction in the control group treated with unlabeled MX35 was 0.15. There was no significant drop in white blood cell counts or weight loss.	Ovarian cancer	[84]
^213^Bi-DTPA-PAN-622-mAb	A pilot therapy study with ^213^Bi-DTPA-PAN-622 demonstrated a significant effect on the primary tumor.	Breast cancer	[85]
^213^Bi-Anti-hCD138 Antibody	TAT of 7.4 MBq and 11.1 MBq significantly improved survival (*p* = 0.0303 and *p* = 0.0070, respectively), whereas HIPEC and HIPEC + TAT treatments did not significantly ameliorate survival as compared with the control group.	Ovarian cancer	[86]
^213^Bi-DOTA-9E7.4-mAb	TAT with 3.7 MBq of ^213^Bi-labeled 9E7.4 anti-CD138 mAb increased median survival to 80 days compared with 37 days in the untreated control group and resulted in effected cure in 45% of the animals.	Multiple myeloma (MM)	[87]
^213^Bi-anti-EGFR-mAb	Treatment with ^213^Bi-anti-EGFR-mAb resulted in an effective induction of cell death in EJ28Luc and LN18 cells.	Bladder carcinoma	[88]
^213^Bi-CHX-A’’-DTPA-anti-CD138-mAb	The combined treatment resulted in significant tumor growth suppression and improved survival in the animals.	MM	[89]
^213^Bi-DTPA-anti-CD38-MAb	Treatment with ^213^Bi-anti-CD38-mAb suppressed tumor growth in myeloma xenografts by inducing apoptosis in tumor tissue and significantly extended survival relative to controls.	MM	[90]
^213^Bi-DTPA-Cetuximab	^213^Bi-cetuximab was found to be significantly more effective in the BRCA-1-mutated triple negative breast cancer (TNBC) cell line HCC1937 than BRCA-1-competent TNBC cell MDA-MB-231. siRNA knockdown of BRCA-1 or DNA-dependent protein kinase, catalytic subunit (DNA-PKcs), a key gene in non-homologous end-joining DSB repair pathway, also sensitized TNBC cells to ^213^Bi-cetuximab.	Breast cancer	[91]
^213^Bi-DTPA-anti-CD20-mAb	In CD20-expressing sensitive as well as chemoresistant, beta-radiation resistant, and gamma-radiation resistant NHL cells, ^213^Bi-anti-CD20 induced apoptosis; activated caspase-3, caspase-2, and caspase-9; and cleaved PARP.	Non-Hodgkin lymphoma	[92]
^213^Bi-DOTA-biotin	Treated with anti-CD45 Ab-SA conjugate followed by 29.6 MBq of ^213^Bi- or ^90^Y-DOTA-biotin, 80% and 20% of mice survived leukemia-free for more than 100 days with limited toxicity, respectively.	Myeloid leukemia	[93]
^213^Bi-DTPA-C595-mAb and ^213^Bi-DTPA-PAI2-mAb	After 16 weeks, systemic injections of ^213^Bi-conjugate at doses of 111, 222, and 333 MBq/kg induced significant tumor growth delay in a dose-dependent manner, compared with the non-specific control at 333 MBq/kg.	Pancreatic cancer	[94]
^213^Bi-DOTA-biotin	Mice injected with anti-CD20 PTRNT or 22.2 MBq ^213^Bi-DOTA-biotin had significantly slower tumor growth than controls (mean tumor volume 0.01 ± 0.02 vs. 203.38 ± 83.03 mm^3^ after 19 days, respectively).	Non-Hodgkin lymphoma	[95]
^213^Bi-CHX-A”-DTPA-7.16.4-mAb	In the same animal model, ^213^Bi radiolabeled immunoliposomes were successful in treating early-stage micrometastases, with median survival times comparable with those obtained with antibody-mediated ^213^Bi delivery.	Breast cancer	[96]
^213^Bi-CHX-A”-DTPA-HuCC49ΔCH2	The median survival time after treatment with ^213^Bi-HuCC49ΔCH2 was 45 days, which was equivalent to the median survival time after treatment with ^213^Bi-trastuzumab.	Colon carcinoma	[69]
^213^Bi (^213^Bi-DTPA-[F3]_2_)	Except for the kidneys, where ^213^Bi-DTPA-[F3]_2_ was present due to renal excretion, ^213^Bi-DTPA-[F3]_2_ accumulated significantly in tumors, but only low activities were found in control organs.	Peritoneal carcinomatosis	[97]
^213^Bi-DTPA-2Rs15d sdAb	Median survival significantly increased when ^213^Bi-DTPA-2Rs15d was given alone or in combination with trastuzumab.	Ovarian cancer	[5]
^213^Bi-DTPA-PAI2-mAb	At 2 days and 2 weeks after cell inoculation, no lymphatic cancer spread was observed in the 222 MBq/kg ^213^Bi-DTPA-PAI2-mAb treated class.	Prostate cancer	[98]

**Table 4:** Overview of Clinical Studies with ^213^Bi-labeled Ligands.

Cancer Type	Radioligand	Patients	Reference
Leukemia	^213^Bi-anti-CD33-mAb (SA)	49	[72]
Melanoma	^213^Bi-anti-MCSP-mAb (SA)	54	[110,114,115]
Glioma	^213^Bi-Substance P (SA)	68	[74,75,76,116]
Bladder cancer	^213^Bi-anti-EGFR-mAb (LR)	12	[78]
Neuroendocrine tumor	^213^Bi-DOTATOC (SA)	25	[19]
mCRPCa	^213^Bi-PSMA-617	1	[80]

SA = systematic administration, LR = locoregional administration.

## Data Availability

Data sharing not applicable: No new data were created or analyzed in this study. Data sharing is not applicable to this article.
